# Neural stem cells: Brain building blocks and beyond

**DOI:** 10.3109/03009734.2012.665096

**Published:** 2012-04-19

**Authors:** Tobias Bergström, Karin Forsberg-Nilsson

**Affiliations:** Department of Immunology, Genetics and Pathology, Science for Life Laboratory, Uppsala University, 751 85 Uppsala, Sweden

**Keywords:** Brain tumors, neural stem cell, tumor biology

## Abstract

Neural stem cells are the origins of neurons and glia and generate all the differentiated neural cells of the mammalian central nervous system via the formation of intermediate precursors. Although less frequent, neural stem cells persevere in the postnatal brain where they generate neurons and glia. Adult neurogenesis occurs throughout life in a few limited brain regions. Regulation of neural stem cell number during central nervous system development and in adult life is associated with rigorous control. Failure in this regulation may lead to e.g. brain malformation, impaired learning and memory, or tumor development. Signaling pathways that are perturbed in glioma are the same that are important for neural stem cell self-renewal, differentiation, survival, and migration. The heterogeneity of human gliomas has impeded efficient treatment, but detailed molecular characterization together with novel stem cell-like glioma cell models that reflect the original tumor gives opportunities for research into new therapies. The observation that neural stem cells can be isolated and expanded *in vitro* has opened new avenues for medical research, with the hope that they could be used to compensate the loss of cells that features in several severe neurological diseases. Multipotent neural stem cells can be isolated from the embryonic and adult brain and maintained in culture in a defined medium. In addition, neural stem cells can be derived from embryonic stem cells and induced pluripotent stem cells by *in vitro* differentiation, thus adding to available models to study stem cells in health and disease.

## One stem cell—multiple progeny

The question whether there is one or several stem cells for neurons and glia attracted the interest of scientists for many years. Theories about the cells of origin for neural cells started to emerge during the second part of the nineteenth century when Wilhelm His (1) suggested that so-called germinal cells gave rise to neurons while glial cells came from what he termed spongioblasts, i.e. that there were two different precursors. This was disputed by Shaper (2) who proposed that germinal cells and spongioblasts were different phases for the same cell and thus hypothesized that a single progenitor cell was the origin of both neurons and glia. None of these theories could be proven right at that time, and the neural stem cell identity was not firmly verified until the 1990s (3).

The complexity of the developing central nervous system (CNS) can be viewed in the light of the large numbers of differentiated phenotypes, including a vast diversity of neuronal subtypes that are generated during ontogeny. For a long time it was argued that since neurogenesis occurs mainly from embryonic day (E) 9–10 in mouse and gliogenesis starts around E16, and continues into postnatal life, the cell types would originate from separate progenitors. However, as lineage analysis showed that neurons and glia arise from a common progenitor in the developing CNS, the concept of a neural stem cell became established. By definition, a neural stem cell is able to self-renew while retaining the capacity to generate neurons, astrocytes, and oligodendrocytes. The three major cell types of the CNS all develop from neuroepithelial cells that populate the neural tube early in mammalian development.

## Neural stem cells in development

Development of the vertebrate CNS starts with invagination of the neural plate to form the neural tube, which originally consists of one layer of neuroepithelial cells. When the neural tube matures, the cellular architecture becomes stratified and neural stem cells are found in the ventricular layer, closest to the lumen, while the post-mitotic cells migrate radially toward the brain surface (4). This organization, usually studied with regard to development of the cerebral cortex, is kept during embryogenesis such that proliferating cells are found in the germinal zones lining the ventricles, and mature offspring migrate to take up their final destinations. The same principle applies to several other brain regions.

**Figure 1.**
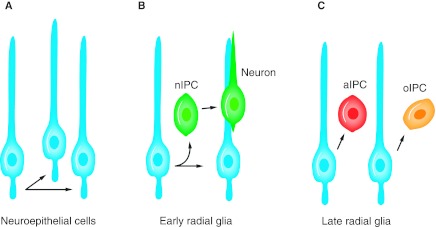
Neuroepithelial cells and radial glia are neural stem cells. nIPC, aIPC and oIPC denote intermediate progenitor cells for neurons, astrocytes, and oligodendrocytes, respectively.

Cortical development, which has been studied in great detail (5), first relies on symmetric cell division, i.e. a neural stem cell divides into two identical cells, thereby allowing the extensive expansion that is needed to build the mammalian brain. Asymmetric cell division prevails during neurogenesis, which starts around E9–10 in the mouse, and results in two distinct cell types: one stem cell and one immature neuron or intermediate progenitor cell (IPC) (also called basic progenitor). Immature neurons migrate away from the ventricular zone and become mature neurons of the cortical plate, whereas the intermediate progenitors reside in the subventricular zone, where they continue to divide and constitute an important reservoir for new neurons throughout neurogenesis. The intermediate progenitors are also able to divide symmetrically, generating two progenitors or two neurons (6–8) (Figure 1A).

‘Neural stem cell' is a widely used term, but during development these building blocks for neurons and glia change shape and characteristics considerably. Already at the start of neurogenesis, neuroepithelial cells are gradually replaced by radial glia (9). These extend a process from the ventricle all the way to the pial surface, while their soma resides in the periventricular area. Radial glial cells show several astroglial properties and express markers known to the glial lineage, such as RC2 (10). Besides their ability to divide asymmetrically and serving as progenitors of neurons and glia, radia glia constitute a scaffold on which neurons migrate in the developing brain (Figure 1B). In fact, they were previously described as a ‘railroad' structure for neurons on their way to their final destinations (11). Now, however, it is appreciated that they actually are stem cells and similar to neuroepithelial cells. They have a polarized organization and display interkinetic nuclear migration during their cell cycle, although radial glial cells do not extend their nuclear migration all the way to the pial end-feet (8,12). Radial glial cells also have a more restricted potential than neuroepithelial cells, which has been shown by fate mapping. *In vivo* evidence of tripotent neuroepithelial cells was demonstrated by retroviral trace labeling, whereas most labeled radial glia gave rise to a single cell type, i.e. neuron, astrocyte, or oligodendrocyte (Figure 1B, C) (13–15).

Difficult as it first seemed to acknowledge glial cells, traditionally regarded as non-neural ‘glue', as stem cells, evidence accumulated over the years in favor of this view, and it is now established that both embryonic, young postnatal, and adult neural stem cells have characteristics of astrocytes. Terminology thus becomes quite confusing, and it must be kept in mind that only a fraction of astrocytes in the postnatal brain has the ability to function as stem cells. However, no attempts have been made so far to introduce a completely new terminology.

## Adult neural stem cells

The view that the adult brain retains the ability to self-renew some of its neurons, and that this is important for normal functions, has emerged over the last 20 years. The identification of areas of adult neurogenesis described in song-birds (16) and rodents (17,18) was a true breakthrough in neuroscience, and when it became apparent that adult neural stem cells also exist in humans it sparkled the field. Decades ago, Altman and Das (19) proposed that postnatal neurogenesis exists in the postnatal rat hippocampus, but their findings were largely ignored due to the technical inability to conclusively label newborn neurons. It was not until the early 1990s that the formation of new neurons in adult rodent brain became evident (17,18,20).

While the majority of neural stem cells will lose their self-renewal capacity and multipotency with time, two germinal zones remain in the brain throughout adulthood. This was shown to be true also for humans, and within these two regions, the dentate gyrus (DG) of the hippocampus (21) and the subventricular zone (SVZ) of the lateral ventricular wall (22), two developmentally different neural stem niches reside. Neurons generated in the SVZ migrate to the olfactory bulb along a process called the rostral migratory stream, originally shown in rodents (23,24) and more recently also in humans (25). Whether neurogenesis can occur in other regions of the adult mammalian brain is still debated (26,27).

In the most widely accepted model of adult neurogenesis, the neural stem cell is a radial, astrocyte-like, GFAP-positive cell (reviewed in (28)). In the subgranular zone (SGZ) of the dentate gyrus, a non-radial stem cell has also been described (29), thus suggesting that the SGZ harbors two structurally different stem cells. The quiescent stem cells are also called type B cells and give rise to actively proliferating intermediate cells, termed C cells, that when they divide generate neuroblasts, type A cells (30). The immature neuroblasts migrate in chains and develop into mature neurons, mostly GABAergic granule neurons in the olfactory bulb, and dentate granule cells in the hippocampus.

The complex regulation of adult neural stem cells is not fully understood. This is partly due to the lack of exclusive markers labeling stem cells and intermediate progenitor cells. However, the increasing understanding of the micro-milieu in the stem cell niche is a key to delineate the specific signals that govern these processes. Renewable tissue in the adult usually harbors specific niches (31). These specialized areas provide nourishment, structural support, and protection to stem cells that may lay quiescently during long periods. The neurogenic niche is composed of blood vessels, local astrocytes, microglia, ependymal cells, and extracellular matrix (ECM) proteins and proteoglycans (32). B cells (the adult stem cells) can reach the ventricle with their apical process, and thereby be in contact with the cerebrospinal fluid, and are also surrounded by ependymal cells, adding to the complexity of regulation (33). The basal process of B cells contact blood vessels in areas with a less stringent blood brain barrier control, i.e. no pericyte coverage or astrocyte end-feet (34), allowing exchange of various factors. The importance of ECM molecules in the niche emerged from both morphological studies (35) and examination of integrin–laminin interactions (36). Local astrocytes can both offer structural support and secrete regulatory factors (37). Microglia in the SGZ were recently shown to be involved in adult hippocampal neurogenesis through their phagocytic properties (38).

## Applications of neural stem cells

Much attention over the last 20 years has been focused on exploring the potential use of stem cells as therapeutic agents, and many important discoveries have been made to advance the field towards the clinic. To convert stem cell research safely into relevant therapeutics we need precise knowledge about the molecules and signaling pathways that regulate proliferation, differentiation, and migration of neural stem cells. Several attempts are already being made to translate neural stem cell discoveries into the patient. In a phase I/II clinical trial by Stem Cells Inc., chronic spinal cord injury patients have been transplanted with purified human adult neural stem cells. Other examples of clinical trials in the neural stem cell field are Neuralstem's phase I clinical trials for amyotrophic lateral sclerosis patients and ReNeuron's trial of a neural stem cell therapy for disabled stroke patients (http://clinicaltrials.gov) (39). The first FDA-approved clinical trial with human embryonic stem cell-derived cells, oligodendrocyte progenitors for spinal cord injury, started in 2010 but was recently stopped (40).

The intriguing finding that transplanted neural stem cells home to experimental brain tumors in mice and in rats (41) was followed by the observation that normal stem cells can migrate to a tumor (42). Furthermore, neural stem cells have shown a tumor-suppressing activity, and transplantation of neural stem cells together with glioma cells represses tumor formation in mice (42,43). Glioma preferentially affects adults with a peak of onset of 50–70 years, while it is a rare disease in children. Neurogenesis declines in the aging brain (44). It can therefore be speculated that neural stem cells in the young brain somehow counteracts glioma formation. Whether the above observations of stem cell tropism will also hold true for human glioma is currently being tested. A pilot study for recurrent high-grade glioma runs between 2010 and 2012 (http://clinicaltrials.gov/ct2/show/NCT01172964) with the rationale that neural stem cells with genetic modifications that allow them to convert 5-fluorocytosine (5-FC) to the chemotherapy agent 5-FU will be transplanted and deliver the cytotoxic agent to the tumor cells when patients are given 5-FC orally.

## Primary neural stem cell cultures

Neural stem cells have been isolated from mice and rats from various regions and time points during development (45,46) as well as from the SVZ and SGZ in the adult nervous system (17,47,48). A widely used method is to culture neural stem cells as free-floating aggregates called neurospheres. These neurospheres grow in defined serum-free media with the addition of epidermal growth factor (EGF) and fibroblast growth factor-2 (FGF-2), which are necessary for maintaining their self-renewal capacity and multipotency (3,45). Although neurosphere cultures allow the propagation of multipotent cells with self-renewal ability, they are heterogeneous and cannot be used as a measure of stem cell content in a given tissue. Both stem and progenitor cells have been shown to give rise to neurospheres (49,50), and thus sphere formation frequency cannot be translated into stem cell frequency (51). Assay parameters such as cell density and medium composition can greatly affect the outcome and interpretation of the assay (52,53). High plating density can induce aggregation of spheres, and it is desirable to use low plating densities to ensure clonality of the formed neurospheres (54,55). An alternative to neurosphere culture is adherent culture, where cells are more easily monitored and has better access to growth factors (56). Adherent culture regimens produce cultures with less differentiated cells compared to the neurosphere assay, where cell–cell contacts induce differentiation (57).

To avoid the problem with neurosphere aggregation, assays such as the neural colony-forming assay (58) have been developed. Here a semi-solid matrix is used to keep colonies apart, which allows for clonal expansion of cells. Other improvements utilize three-dimensional matrices to enhance culture conditions (59,60). Recently, the effect of oxygen pressure has been acknowledged as an important factor for neural stem cell regulation. Lower-than-atmospheric oxygen pressure has been shown to increase proliferation rates and alter differentiation potencies in neural stem cells (61–63).

## Neural stem cells from embryonic stem cells

Mouse embryonic stem (ES) cells are clonal cell lines derived from pre-implantation embryos. They can be maintained in a pluripotent stage in culture (64,65) and, when injected into the inner cell mass of blastocyst stage embryos, contribute to the embryo and can populate all lineages including the germ line (66). This property has been extensively used for the purpose of genetic targeting of mice (67), and both ES cells and the knock-out mouse technology have been awarded the Nobel Prize (http://www.nobelprize.org/nobel_prizes/medicine/laureates/2007/). ES cells have also shown a tremendous potential as *in vitro* models due to their extraordinary capacity to generate different cell types in culture (68).

For the study of brain development, *in vitro* differentiation of ES cell can serve dual purposes. Firstly, when inactivation of a gene leads to an embryonic lethal phenotype, its causes can be examined *in vitro*. Secondly, their unlimited capacity to replicate make ES cells an ideal tool for the step-wise generation of neural progenitors and subsequently neurons and glia needed in cell models of nervous system development. For *in vitro* maintenance mouse ES cells are usually grown on mouse embryonic fibroblast feeder cells, although feeder-independent lines exist, and pluripotency requires the presence of LIF in the medium. Differentiation is achieved when LIF is discontinued and the cells taken off the feeder layer. A multitude of *in vitro* differentiation protocols for mouse ES cells to various cell lineages have been described (69).

**Figure 2.**
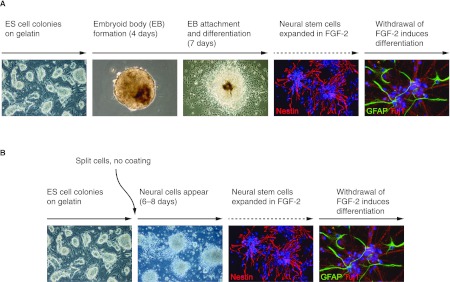
*In vitro* differentiation of ES cells to neural stem cells. A: Neural stem cells via embryoid body formation. B: Neural stem cells via monolayer differentiation.

There are two principally different ways to obtain neural progenitors. The first protocol (Figure 2A) uses formation of embryoid bodies that subsequently adhere to coated plastic surfaces in a defined medium and convert into rosette-like neural cells that are indistinguishable from primary neural stem cells in culture (70). This cell population is highly enriched for neural progenitors and proliferates in neural stem cell medium in the presence of FGF-2. Following withdrawal of the mitogen, differentiation into neurons and glia is achieved. Further maturation generates functional neurons, as determined by electrophysiology (70) and incorporation in the mouse brain after transplantation (71). A second, less cumbersome method (Figure 2B) is the adherent monolayer protocol established by Ying et al. (72) and further developed (73). By this method, embryoid body formation is dispensable, and conversion of ES cells to neural precursor cells occurs within 5–7 days of culture in the neural promoting medium N2B27. A vast majority of the cells express nestin, an intermediate filament present in neural precursor cells, show a neuroepithelial morphology, and can be expanded in FGF-2 with retained potential for differentiation into multiple cell fate choices.

A great breakthrough in stem cell biology was achieved in 1998 when Thomson and colleagues derived the first human ES cell line (74). The hES cells and mouse ES cells are different not only by species barriers but also in that the hES cells are more similar to the recently isolated mouse epiblast stem cells (75,76). Consequently, the culture conditions of hES and mES are different, and it has been necessary to adapt differentiation protocols to hES cells. As with mES cells, spontaneous differentiation to neurons is inefficient, and different specific neural selections procedures are employed. Human ES cells can now be used to study human development, and, for neural differentiation, embryoid body formation or stromal cell co-culture has mostly been used (reviewed in (77)). However, an adherent monolayer protocol similar to that of mouse ES cells was also applied (78). Most recently, a xeno-free protocol for generation of specific neural cells, in this case oligodendrocytes, has been published (79).

## Neural stem cells from induced pluripotent stem cells

The derivation of human induced pluripotent stem cells lines (iPSC) (80,81) opened up new possibilities for studies of human CNS development and disorders. iPSC resemble hES cells with respect to expression of genes, epigenetic modifications, and ability to differentiate into a multitude of progeny (82). Although iPSC retain the epigenetic memory of their original somatic cells (83,84), many characteristics are similar to hES cells, such as pluripotency marker expression, self-renewal, and multilineage potential. Mouse iPSCs also contribute to germline formation, but recently a disturbing possible difference was noted (85), when it was found that autologous mouse iPSCs were rejected by the host immune system. A variety of neural differentiation protocols adopted from hES cells have already been applied to iPSC (86). Furthermore, in a recent study, neurons from patient-derived iPSC were used to investigate the mechanism of a neurodegenerative disorder (87).

The ability to generate patient-specific stem cells using iPS technology gives them great potential in future personalized medicine, but too little is yet known about these cells to make any firm predictions. Knowledge about human ES cells, on the other hand, is more solid, and protocols for clinical grade cell production of xeno-free cultures are already here. A remaining challenge with ES cell-derived grafts, the problem that undifferentiated progenitors are prone to form tumors, has been solved by novel sorting and selection procedures. It seems likely that iPS cells are even more tumorigenic than hES cells in their undifferentiated state (88), and efforts are therefore needed to design *in vitro* differentiation protocols that will exclude tumor formation *in vivo*. Whether hES cells or iPS cells will be the most important tool in stem cell therapies remains to be seen.

## Neural stem cell markers

No single marker exists to label exclusively neural stem cells. Instead a combination of prospective sorting and retrospective analysis of potency and self-renewal capacity is needed to reveal stem cell identities. During early development, neuroepithelial cells are positive for nestin (89) and SOX2 (90). At the onset of neurogenesis, around E9–10 in the mouse, neuroepithelial cells transform into radial glial cells and begin to express astroglial markers such as GLAST, BLPB, and GFAP (only in humans and primates) and the radial glial marker RC2 (10).

In the adult SVZ, neural stem cells (type B cells) express astroglial markers GFAP, GLAST, BLBP, connexin 30, vimentin, and nestin. Also CD133 and Fut4 (also known as LeX/CD15/SSEA-1) have been shown to label neural stem cells in the SVZ. More differentiated C cells express EGFR, Ascl1 (Mash1), and DLX2. Some GFAP-positive type B cells express EGFR, and it has been proposed that the EGFR expression denotes an ‘activated' neural stem cell. Migrating neuroblasts, type A cells, can be distinguished by their expression of DCX and PSA-NCAM. It should be noted that neural stem cells and more restricted progenitors express overlapping sets of these markers, making it difficult to use solely markers to identify a particular cell type (91).

## Maintenance of ‘stemness' properties

Intrinsic and extrinsic signals play important roles for the regulation of neural stem cell fate (3,92). Among the most potent extrinsic regulators are the soluble growth factors EGF and FGF-2, which both support neural stem cell proliferation and self-renewal capacity. Upon withdrawal of EGF and FGF-2, neural stem cells will spontaneously differentiate into a mixture of neurons, astrocytes, and oligodendrocytes. The developmentally important Notch signaling pathway is also central for maintaining neural stem cell proliferation, in part by transcriptionally activating the target genes Hes1 and Hes5 (93,94), which in turn inhibit the pro-neuronal genes Mash and neurogenin2. Notch signaling thus favors a glial and radial glial fate (95).

## Differentiation properties and growth factor responses

A few soluble factors are known efficiently to influence neural stem and progenitor cell fate determination, of which ciliary neurotrophic factor (CNTF) induces astrocytic cell fate (56,96), and the thyroid hormone, tri-iodothyronine (T3) (56), will increase the number of oligodendrocytes formed. Although platelet-derived growth factor (PDGF) was originally suggested to induce neuronal differentiation (56), we subsequently showed that PDGF instead expands progenitors (97) and that if endogenous PDGF is blocked it enhances differentiation mainly to neurons and oligodendrocytes (98). To date, no efficient one-step neuronal extrinsic inducer has been described, although a combination of retinoic acid and forskolin efficiently induced neuronal formation in an adult hippocampal neural stem cell line (48).

However, intrinsic regulators such as the proneural neurogenins (99,100), Wnt--catenin signaling (101), and b-HLH transcription factors are clearly important for the acquisition of a neuronal fate (102). The transcription factor Lmx1 was shown to be sufficient and required for formation of dopaminergic neurons (103). A number of transcription factors that efficiently induce astrocytic and oligodendrocytic fate are also known, including Olig1 and 2, Hes1 and 5, and Ngn3 (104,105).

## Stem cells and brain tumors

Already in 1858, Rudolf Virchow suggested a link between developmental-stage tissue and tumors when he proposed that embryonic cells give rise to cancers (106) due to their histological similarities with tumors. In 1926, Baily and Cushing suggested a classification system for neuron-glia malignancies based on the histogenetic approach (107). Several of the terms that they proposed have been kept and are still used histologically to classify brain neoplasms (108). The most malignant form of glioma, grade IV, or glioblastoma multiforme (GBM) is also the most frequent brain tumor, with an incidence of 3–4 new cases per 100,000 per year, and it preferentially affects adults. GBM can be of either primary type (90% of the cases) and develop rapidly without prior illness, or secondary (the remaining 10%), beginning with a less malignant cancer. Despite advances in research on the molecular mechanisms underlying malignant glioma, these tumors remain fatal with a median survival of 14–15 months (109).

The high proliferative rate, large degree of heterogeneity, and rapid invasion of neoplastic cells into healthy brain tissue are all pathological hallmarks of malignant glioma. A more detailed view of the molecular networks that are perturbed in GBM is starting to emerge due to the integrated genomic and expression data gathered e.g. by the Cancer Genome Atlas (TCGA) (110). Accumulated data show that the principal pathways identified are the RAS/MAPK and P13K/AKT pathways, tumor suppressors TP53, RB, and PTEN. This means that all major ways to control proliferation and survival are affected in GBM. Also, a rapidly expanding amount of data regarding promoter methylation, miRNA expression, and proteome analysis of GBM patients continues to contribute to the knowledge base for future therapies (111).

Indications that neural progenitors could be the origin of primary brain tumors came from biopsy specimens and studies of tumor cell lines (112). Furthermore, primary brain tumors share many characteristics with neural stem/progenitor cells, and a concept of ‘brain tumor stem cells' has emerged (113–116). In a series of experiments, patient-derived neurospheres were shown to possess neural stem cell-like characteristics, such as marker expression, self-renewal capacity, and a propensity to change phenotype and cell surface markers (‘differentiate') in response to differentiating culture conditions. Stem cell-like cells from glioblastoma, grown as neurospheres have been shown to have a greater capacity to self-renew compared to neurospheres from normal brain (117), and cells from tumor-derived spheres readily generate glioblastoma-like tumors in nude mice. Furthermore, key pathways perturbed in glioblastoma are neural stem cell pathways (118), and the transcriptional profiles of neural stem cells and experimental glioma are largely overlapping (119). Thus, stem cells with impaired growth control could become a threat to the organism, and neural stem cells that exist throughout life may accumulate mutations that could result in a transformed progeny. To investigate the relationship between stem cells and brain tumors, new cell models based on stem cell culture conditions for glioma patient biopsies have emerged (120,121). In collaboration with L. Uhrbom's and B. Westermark's laboratories we are generating a cell bank for glioblastoma cell lines in defined, serum-free medium which are expected, at least partly, to maintain the properties related to the original tumor.

**Figure 3.**
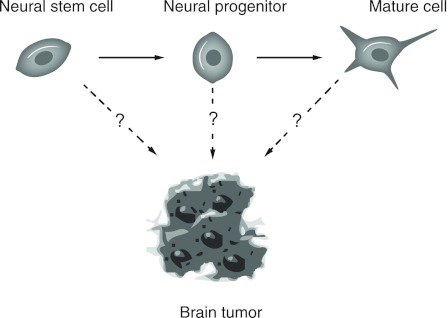
Cells of the neural lineage as possible brain tumor-initiating cells.

The concept of cancer stem cells, which was adapted to brain tumors from the hematopoietic tumors was originally proposed for acute myeloid leukemia (122). It proposes that cancers are driven, not by the bulk of the tumor cells but by a small subpopulation, the cancer stem cells. However, tumor growth need not only be caused by rare cancer stem cells (123,124), and it remains unclear whether it is the stem cell or an intermediate progenitor that is the tumor-originating cell (Figure 3). Because adult neural stem cells divide slowly, while their progeny display rapid cell cycle progression, the term cancer progenitor might be more appropriate. Elaborate experiments, rarely undertaken, are needed to ensure that observations made are due to the stem cells rather than a partly restricted progenitor. Therefore, the identity of the cell of origin for malignant glioma remains elusive. It could either be a stem cell, a progenitor cell, or a specialized cell type undergoing dedifferentiation.

## Concluding remarks

Since the 1990s, neural stem cells have been proven as a concept, and we have seen the expansion of a whole research field. The concept of postnatal neurogenesis has made its way into textbooks, overthrowing the old dogma that no new neurons are formed in adult life. In fact, adult neurogenesis is even more relevant for daily life than we would ever have dreamt of. This is exemplified by findings that hippocampal neurogenesis plays an important role in learning, buffering stress response, and depression, and that physical activity and a stimulating environment can have a positive effect on neurogenesis. Most of the above findings are based on animal models, but an increasing amount of information regarding human neurogenesis is being gathered.

Stem cell proliferation and differentiation can be regarded as a balance, with a risk that unlimited growth due to perturbations in growth regulatory pathways can lead to tumor formation. The link between neural stem cells and malignant brain tumors has become more firmly established over the years, but we still do not know the exact role of stem cells versus more mature progenitors and differentiated cell types in cancer initiation and progression. The challenge is the poor prognosis for glioblastoma patients, which has only been marginally improved. New tools, such as well-defined patient-derived glioblastoma cell lines grown in stem cell culture conditions, will be useful to test new therapeutic approaches.

Neural stem cells have also proven extremely valuable as disease models, and the results from animal models are now tested in a first set of clinical trials, both for neurodegenerative disorders and recurrent high-grade glioma. These are exciting times for neural stem cell biology, but drawbacks may also occur that can halt the on-going translation of stem cell biology to the clinic. Regardless of the time needed for advancement of stem cell medicine, knowledge generated from studies of neural stem cells has greatly expanded our understanding of how the central nervous system develops. In fact, neural stem cell biology is now a mature research field and can today be regarded as an integrated part of neurobiology.
